# Effect of Stress Aging on Strength, Toughness and Corrosion Resistance of Al-10Zn-3Mg-3Cu Alloy

**DOI:** 10.3390/ma18010181

**Published:** 2025-01-03

**Authors:** Dongchu Yang, Xi Zhao, Xianwei Ren, Shiliang Yan, Yihan Gao, Hongbin Liu

**Affiliations:** 1 School of Aerospace Engineering, North University of China, Taiyuan 030051, China; er69871394@163.com (D.Y.); yslll583797428@163.com (S.Y.); yhgao@nuc.edu.cn (Y.G.); 2 Engineering Technology Research Center for Integrated Precision Forming of Shanxi Province, North University of China, Taiyuan 030051, China; 3 Shandong Zhuoyue Precision Industry Group Co., Ltd., Jining 272114, China; 19834409076@163.com

**Keywords:** stress aging, strength, toughness, corrosion resistance, Al-Zn-Mg-Cu alloy

## Abstract

The 7000 series aluminum alloy represented by Al-Zn-Mg-Cu has good strength and toughness and is widely used in the aerospace field. However, its high Zn content results in poor corrosion resistance, limiting its application in other fields. In order to achieve the synergistic improvement of both strength and corrosion resistance, this study examines the response of strength, toughness and corrosion resistance of a high-strength aluminum alloy tail frame under aging conditions with external stresses of 135 MPa, 270 MPa and 450 MPa. The results show that with the increase in the external stress level, the strength of the alloy improves, while its corrosion resistance decreases. An optimal balance of strength, toughness and corrosion resistance is achieved at the conditions of 270 MPa-120–24 h. This phenomenon can be attributed to two main factors: first, lattice defects such as vacancy and dislocation are introduced into the stress aging process. The introduction of a vacancy makes it easier for neighboring solute atoms to migrate there. This makes the crystal precipitates more dispersed. Also, the number of precipitates in the matrix increases from 2650 to 3117, and the size is refined from 2.96 nm to 2.64 nm. At the same time, the dislocation entanglement within the crystal structure promotes the dislocation strengthening mechanism and promotes the solute atoms to have enough channels for migration. Since too many dislocations can cause the crystal to become brittle and thus reduce its strength, entangled dislocations hinder the movement of the dislocations, thereby increasing the strength of the alloy. Secondly, under the action of external force, the precipitated phase is discontinuous, which hinders the corrosion expansion at the grain boundary, thus improving the corrosion resistance of the alloy. At low-stress states, the binding force of vacancy is stronger, the precipitation free zone (PFZ) is significantly inhibited, and the intermittent distribution effect of intergranular precipitates is the most obvious. As a result, the self-corrosion current decreases from 1.508 × 10^−4^ A∙cm^−2^ in the non-stress state to 1.999 × 10^−5^ A∙cm^−2^, representing an order of magnitude improvement. Additionally, the maximum depth of intergranular corrosion is reduced from 274.9 μm in the non-stress state to 237.7 μm.

## 1. Introduction

As the aerospace and marine industries evolve, there is a growing need for lightweight, high-performance parts, along with increased demands for corrosion resistance. Al-Zn-Mg-Cu alloys are frequently utilized to fulfill the requirement for lightweight structures because of their excellent strength and low weight [1,2,3,4,5]. Researchers have enhanced the mechanical properties of components by augmenting the zinc content and employing techniques such as upsetting, extrusion, and reverse extrusion forming, resulting in an increase in strength to levels ranging from 600 to 700 MPa, and in some cases, up to 800 MPa [6,7,8]. The main strengthening mechanism is to promote the precipitation of the metastable η’ phase while inhibiting the stable η phase [9,10] An increased zinc content not only facilitates the dispersion of the precipitated phase within the grains but also promotes a continuous distribution of the precipitated phase along the grain boundaries [11,12,13]. This phenomenon adversely affects corrosion resistance and limits the applicability of Al-Zn-Mg-Cu alloys.

To address the need for the coordinated improvement of both the mechanical properties and corrosion resistance of Al-Zn-Mg-Cu alloys, extensive research has been conducted, with most focusing on regression and re-aging (RRA) treatment. These studies all show that the T6-treated components will maintain high strength and plasticity but show weak corrosion resistance in this state [6,7,8,9,10,11,12,13,14,15,16]. While over-aging treatment can effectively improve the corrosion resistance of the alloy, it will also lead to a reduction in its strength [17,18,19]. At the same time, the RRA process requires the components to be treated to be exposed at high temperatures for a short time, so it is not suitable for complex components [20,21]. As conventional aging treatment makes it difficult to meet scholars’ expectations, and based on the complex and highly integrated forming technology needs of large-size passenger aircraft skin, the focus is turned to a new integrated forming technology, namely creep forming. Creep forming is mainly used to combine the artificial aging of materials with the forming of parts, and at the same time, the creep deformation of materials under the action of elastic stress at a certain temperature is used to obtain structural parts with a certain shape. Based on creep aging forming technology, stress aging has gradually become a research hotspot in the field.

Stress aging is usually applied during the aging process, with stress not exceeding the yield strength. A substantial body of research has demonstrated that the application of external stress during the artificial aging process markedly influences both the nature and dimensions of precipitates [22,23,24,25,26,27,28]. Quan et al. [29] showed that the application of uniaxial stress can improve the mechanical properties of the alloy while reducing the time to peak strength. Increasing applied stress tends to accelerate the growth of precipitates, while the growth and intermittent distribution of grain boundary precipitates (GBPs) in turn enhance corrosion resistance. Guo et al. [27] posited that the alterations observed in the precipitated phase in response to external stress can be ascribed to the significant quantity of defects generated by the stress, which subsequently modified the diffusion rates of solute atoms and vacancies. While the diffusion rate of solute atoms decreases, due to the vacancy introduced by stress, the nucleation sites increase, and there is a strong competitive relationship between phase growth, which inhibits the growth of the precipitated phase and makes it difficult for the strengthened phase η ’to transform into the stable phase η. At the same time, fewer solute atoms migrate to the grain boundary, resulting in a discontinuous distribution of GBPs. Lin et al. [30,31] have indicated that the process of stress aging can enhance the refinement of aging precipitates. Furthermore, they noted that the application of external stress tends to decrease the width of the precipitate-free zone (PFZ). Zhang et al. [32] noted that the application of compressive stress can enhance the migration of solute atoms. Nevertheless, the constant total quantity of solute atoms establishes a competitive dynamic that inhibits the coarsening of the precipitate phase. This competitive growth refines the precipitated phase and improves the mechanical properties of the alloy.

In summary, unlike the traditional RRA processing method, this paper focuses on the stress-aging process to regulate the types, morphologies, and sizes of precipitates, thereby achieving a coordinated enhancement of the mechanical properties and corrosion resistance of Al-Zn-Mg-Cu alloys. In this study, the effect of applied new stress on the number and size of intergranular phases was systematically studied by using newly developed engineering samples, and the strengthening and toughening mechanism and the effect of applied stress on grain boundary phases were elucidated. By combining potentiodynamic polarization, slow strain rate testing (SSRT) and transmission electron microscopy (TEM), this study examines the mechanism of stress on the mechanical properties and corrosion resistance of Al-Zn-Mg-Cu alloy, providing guidance for the engineering application of new Al-10Zn-3Mg-3Cu alloy barrel components.

## 2. Material and Experiments

### 2.1. Material and Aging Treatment

**Figure 1:**
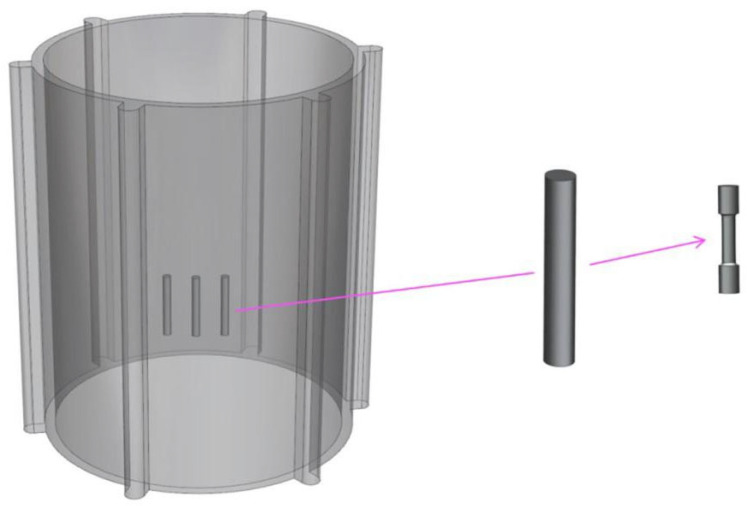
Sample sampling diagram.

The material selected for this experiment is a newly developed aluminum alloy from Central South University. The composition of the Al-Zn-Mg-Cu alloy is shown in Table 1 below, and the sampling diagram is shown in Figure 1.

**Figure 2:**
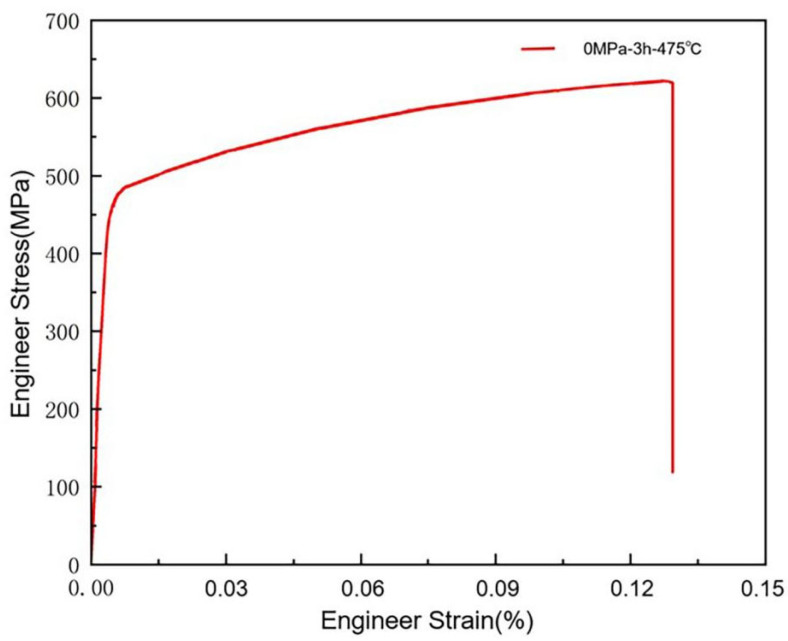
Mechanical properties in S0 state.

In this study, the sample was first a solution treatment at 475 °C for 3 h, after which it was formed into a tensile rod with a diameter of 5 mm. After the solid solution treatment, tensile tests were conducted on the sample using an Instron3382 universal tensile testing machine (Shenzhen, China). The yield strength (YS) and ultimate tensile strength (UTS) of the sample were measured to be 450 MPa and 624 MPa, respectively, with an elongation of 12.5%, as shown in Figure 2. In order to test the changes in the strength, toughness and corrosion properties of the sample after stress-aging and peak-aging treatments, a stress-aging time of 24 h was selected. Based on the yield strength tested above, three groups of stress levels were set: 0.3$\sigma_{0.2}$, 0.6$\sigma_{0.2}$ and $\sigma_{0.2}$, respectively. The final testing scheme is detailed in Table 2. Then, the experimental scheme was designed, and the creep stress testing machine of Central South University was used for stress aging. After stress aging, a tensile test, transmission electron microscope scanning and a corrosion test were carried out to test the mechanical properties, microstructure and corrosion resistance of the samples. The design of the tensile experiment, transmission electron microscope scanning and corrosion experiment is shown below.

### 2.2. Properties Testing

The tensile sample, as described in Table 2, was subjected to tensile testing to measure the yield strength, tensile strength and elongation of the sample after stress aging. The tensile rate in the tensile test was set to 0.5 mm/s. Three measurements were made in the test, and the average values were taken.

### 2.3. Microstructural Investigations

**Figure 3:**
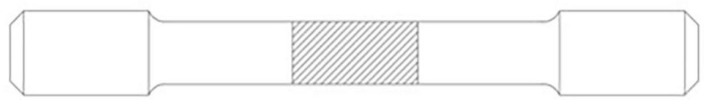
Schematic diagram of TEM sample sampling.

The microstructure of the samples treated in Table 2 was examined using transmission electron microscopy (TEM) (Shanghai, China). TEM samples were prepared by extracting a small part of the sample (the shaded area shown in Figure 3), grinding it to a thickness of 80 μm, electropolishing with a double-jet system in a solution of 30%HNO_3_ + 70%CH_3_OH (China), and finally conducting ion thinning. The microstructure was then observed under the transmission electron microscope. Its instruments are from the University of Science and Technology Beijing (Beijing, China).

### 2.4. Corrosion Testing

In order to test the corrosion resistance of the sample treated as described in Table 2, both an intergranular corrosion (IGC) test and an electrochemical corrosion test were performed. According to the latest intergranular corrosion experimental criteria in the field, the IGC test exposed the sample to a corrosive medium for 6 h according to GB/T 7998-2005, where the surface-area-to-solution-volume ratio was 5 mm^2^/mL. Multiple measurements were taken, and the maximum corrosion depth was taken to compare the corrosion resistance between different experiments. The polarization curve was measured through electrochemical experiments. The working area of the alloy sample was 0.5 cm^2^, and the corrosion medium was a 3.5wt% sodium chloride solution. The experiment was conducted using a CHI760E (Jinan, China) electrochemical workstation, with an Ag/AgCl reference electrode. In the experiment, the open circuit potential (OCP) was measured first, followed by the polarization curve. The scanning rate was set to 3 mV/s, with a scanning range of OCP ± 700 mV.

## 3. Results

### 3.1. Tensile Property

**Figure 4:**
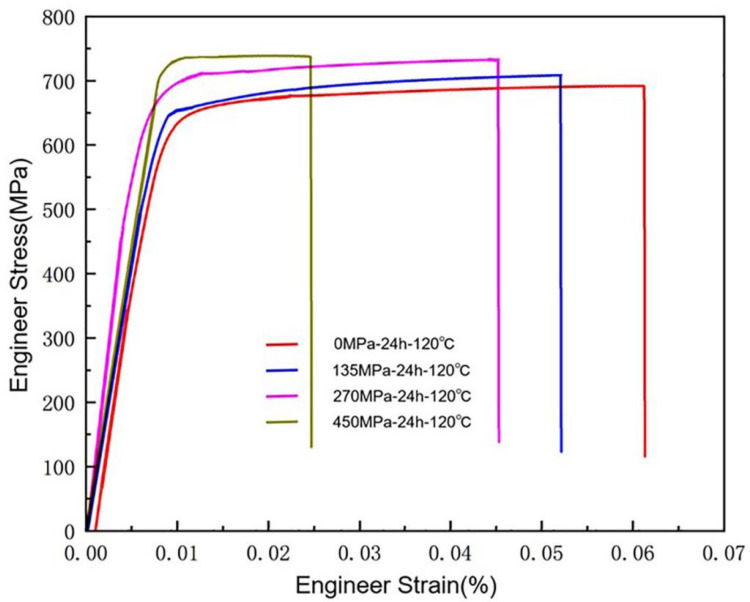
Changes in material properties after different aging treatments.

Figure 4 shows the mechanical properties of the materials after different aging treatments, as measured through tensile testing. Table 3 shows the mechanical properties of the sample under different aging conditions obtained from Figure 4. In comparison to the S0, the mechanical properties of the alloy exhibit considerable enhancement following peak aging and stress aging. Following the peak-aging treatment, the application of external stress resulted in an enhancement of the strength of the sample. YS and tensile strength of the sample in its peak-aging condition exhibited increases of 42.2% and 10.9%, respectively, in comparison to the solid solution state. In the case of the S135, the tensile strength demonstrated an approximate increase of 2.89% relative to the peak-aging state, whereas the yield strength remained constant. This observation suggests that the application of a stress level of 135 MPa does not substantially enhance the mechanical properties of the sample. When observing S270, it was found that YS and UTS exhibited enhancements of 8.6% and 6.1%, respectively, in comparison to the SF. At 450 MPa, YS and UTS further increased by 11.4% and 7.1%, respectively. The experiments show that the elongation of the alloy decreases with the increase in mechanical properties. Comparing the S270 and S450, YS and UTS at 450MPa were 2.6% and 1% higher, respectively. Therefore, the order of strength after aging treatment was: S450 > S270 > S135 > SF > S0, while the elongation ranking is: S450 < S270 < S135 < SF < S0. These changes in properties may be due to the presence of external stress to introduce defects, the existence of defects will inhibit the growth of precipitated phase but also hinder the deformation of the crystal. This makes the mechanical properties of the material improve but the elongation decreases. The more such defects, the worse the elongation performance.

### 3.2. Intergranular Corrosion Resistance

**Figure 5:**
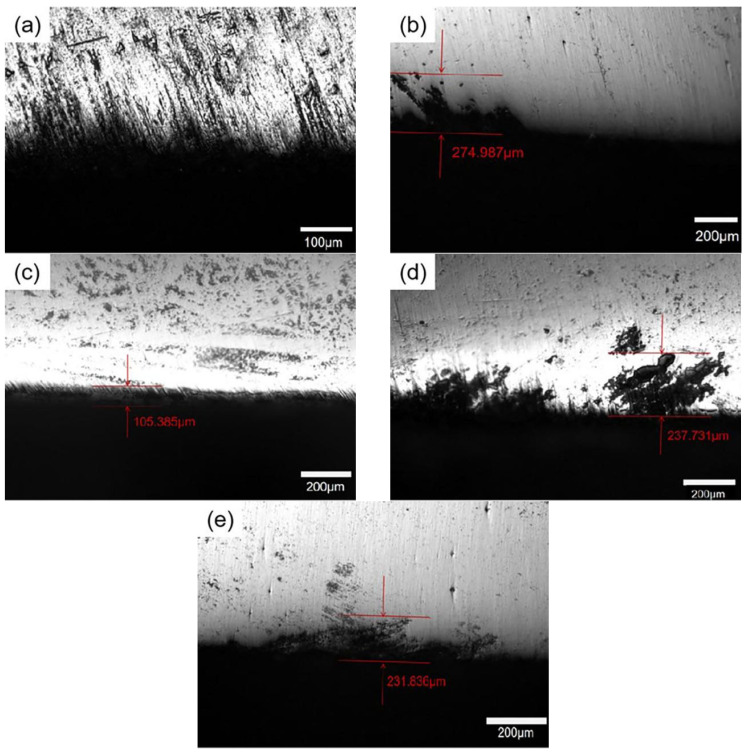
Intergranular corrosion of alloys after different aging treatments: (**a**) S0; (**b**) SF; (**c**) S135; (**d**) S270; (**e**) S450.

The processes of aging heat treatment and stress aging exert a considerable influence on the corrosion resistance of the material. As shown in Figure 5, the sample’s corrosion after being soaked for 6h in the corrosion solution required in GB/T 7998-2005 (57 g NaCl + 10 mL H_2_O_2_ + 1 L deionized water) varied depending on the treatment. In the solid solution state, there was no significant evidence of intergranular corrosion. However, compared to S0, the corrosion of the SF alloy deteriorates, and the sample exhibits the lowest corrosion resistance. While intergranular corrosion was still present in the samples after undergoing stress-aging treatment, its severity was notably reduced compared to that observed in the peak-aging condition. Metallographic analysis of cross-sections revealed the maximum corrosion depth: 274.9 μm at peak-aging state, 105.4 μm under 135 MPa stress, and 237.7 μm under 270 MPa stress, and 231.8 μm under 450 MPa stress. It can be obviously seen that the corrosion resistance of the sample is improved after stress aging, and the maximum corrosion depth of S135 alloy is the lowest. As a result of external stress, the corrosion resistance of the sample is enhanced.

### 3.3. Electrochemical Corrosion Behavior

**Figure 6:**
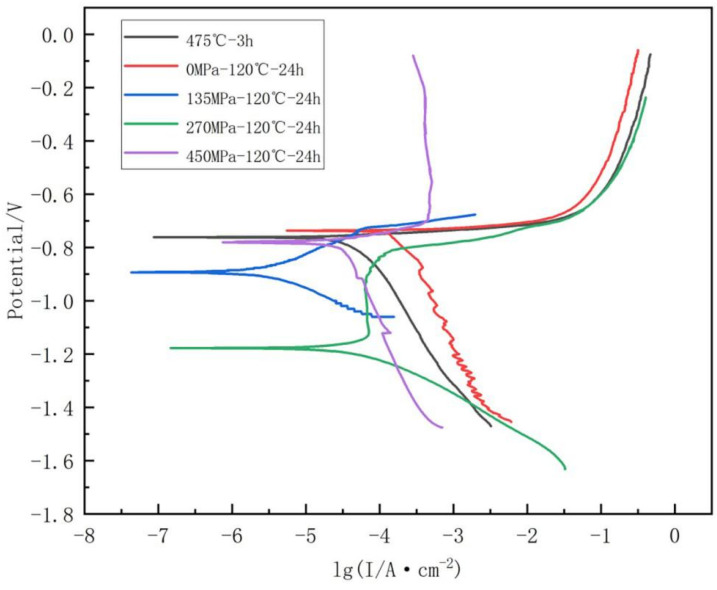
Polarization curves under different levels of stress aging.

Figure 6 illustrates the polarization curves of different samples. The results indicate that all samples exhibited a comparable trend. The self-corrosion potential (Ecorr) and corrosion current density (Icorr) were determined using standard extrapolation techniques. According to Figure 6, the parameters can be obtained by extrapolation, which are summarized in Table 4.

It can be clearly found from Table 4 that the electrochemical parameters change with different aging treatments. With the increase in aging time, an increase in Ecorr indicates a decreasing tendency for corrosion, while a decrease in Icorr suggests a slowing corrosion rate. After aging, significant changes in Ecorr and Icorr were observed. Compared with the solution-treated alloy (Ecorr = 762 mV), the Ecorr of the alloy after peak-aging treatment was reduced to 737 mV. In contrast, the Ecorr values for the S135, S270, and S450 stress-aged samples were 894 mV, 1178 mV, and 781 mV, respectively. The Icorr value of the S0 is 2.583 × 10^−5^ A∙cm^−2^, the Icorr value after peak-aging treatment is 1.508 × 10^−4^ A∙cm^−2^, and the Icorr treated with S135 is 2.962 × 10^−6^ A∙cm^−2^. The Icorr of the alloy treated with S270 is 1.999 × 10^−5^ A∙cm^−2^, and the Icorr of the alloy treated with S450 is 8.892 × 10^−6^ A∙cm^−2^. According to Faraday’s Law [33,34], the self-corrosion rate of the sample increases with the increase in Icorr value. Therefore, Ecorr and Icorr values should be considered in the evaluation of local corrosion behavior. According to the data, it can be seen that the Ecorr value of the S135, S270 and S450 aging treatments is higher than SF, and the Icorr value is lower than SF. Meanwhile, compared with the corrosion condition of intergranular corrosion, we found that the samples treated with applied stress aging showed better corrosion resistance.

### 3.4. Precipitated Phase Characterization

**Figure 7:**
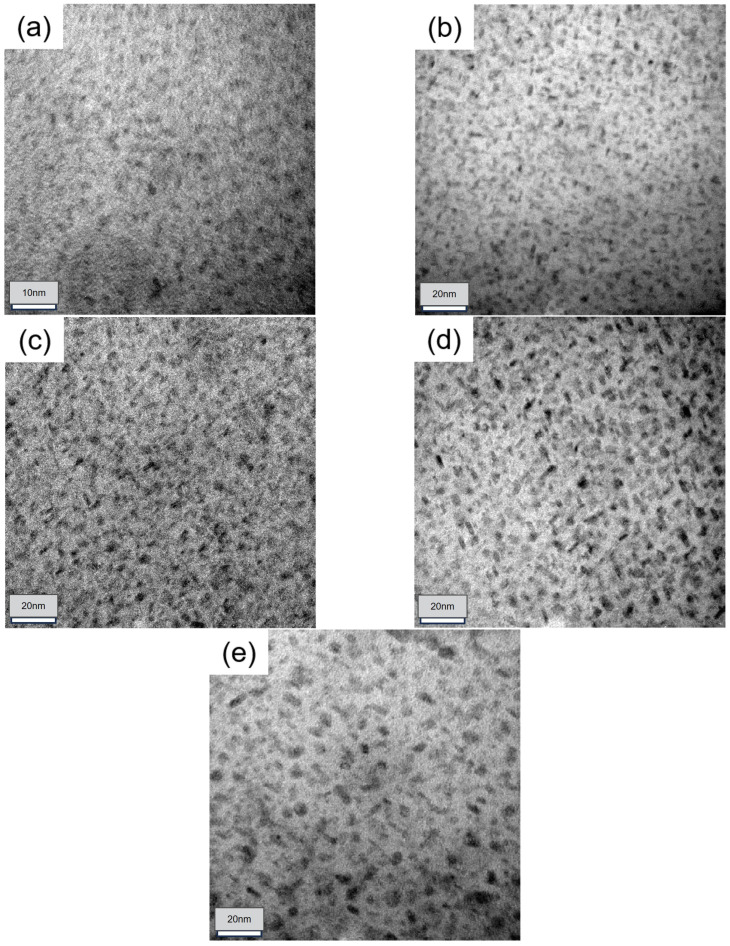
TEM bright field image in alloy: (**a**) S0; (**b**) SF; (**c**) S135; (**d**) S270; (**e**) S450.

The changes of the mechanical properties and corrosion resistance of the samples under different aging treatments were investigated by analyzing their microstructural evolution, as summarized in Table 2. The microstructure of the sample after different aging treatments was compared and observed using TEM bright-field (BF) imaging along the <110>Al axis (Figure 7). BF images indicate that the morphological characteristics of microplastics (MPs) vary across different aging states. In particle size calculation, MPs of different shapes are approximated as spheres of equal diameter for convenience of statistics. The average sizes were measured using a Nano Measurer, and then the MP number was quantified using Image Pro+.

**Figure 8:**
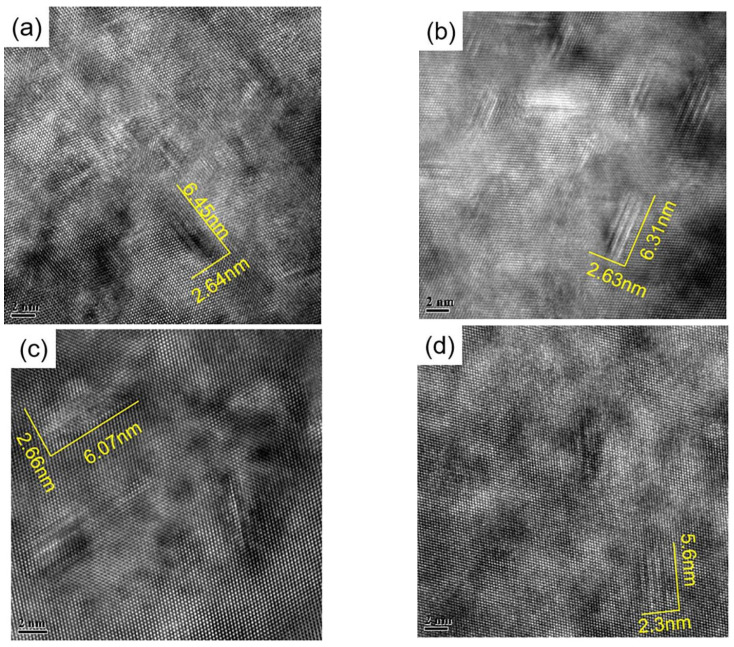
HRTEM image of alloy along <110>Al axis: (**a**) SF; (**b**) S135; (**c**) S270; (**d**) S450.

**Figure 9:**
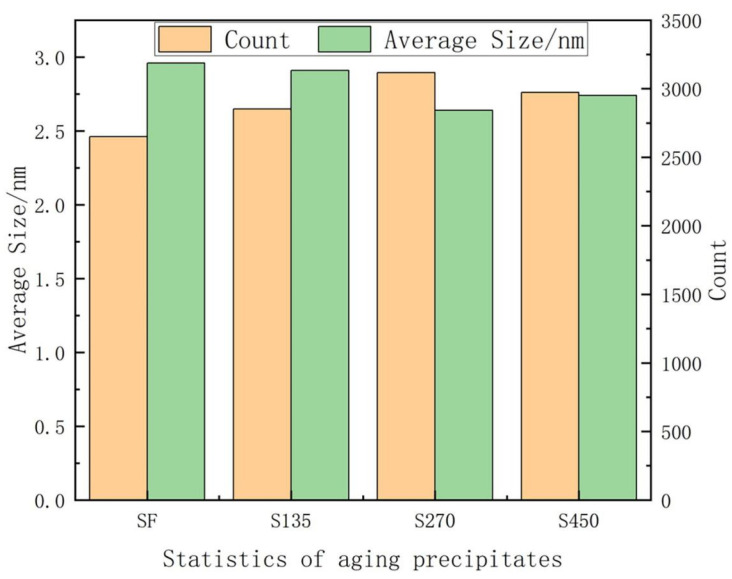
Statistics of aging precipitates.

For the alloy treated with solid solution, as shown in Figure 7a, very few precipitates were observed, and minimal precipitation was seen at the grain boundaries, rendering the PFZ at the grain boundary negligible compared with other samples. After aging treatment under the SF condition, the quantity and density of the precipitated phase changed greatly, and the amount of these fine precipitated phases in the crystal is obviously increased. It can be observed from the TEM images that the density of the internal precipitated phases in the alloy samples after aging treatment with external stress is further increased, and the size of the grains is further reduced. As shown in Figure 8, the sizes of precipitated phases in samples SF, S135, S270 and S450 are 6.45 nm, 6.31 nm, 6.07 nm and 5.6 nm, respectively. This result is similar to the data measured in Figure 9. Moreover, the mechanical properties of the alloy are further improved after the applied stress, because the number of η’ phases increases and the size decreases, so more fine η’ phases can improve the mechanical properties of the alloy.

**Figure 10:**
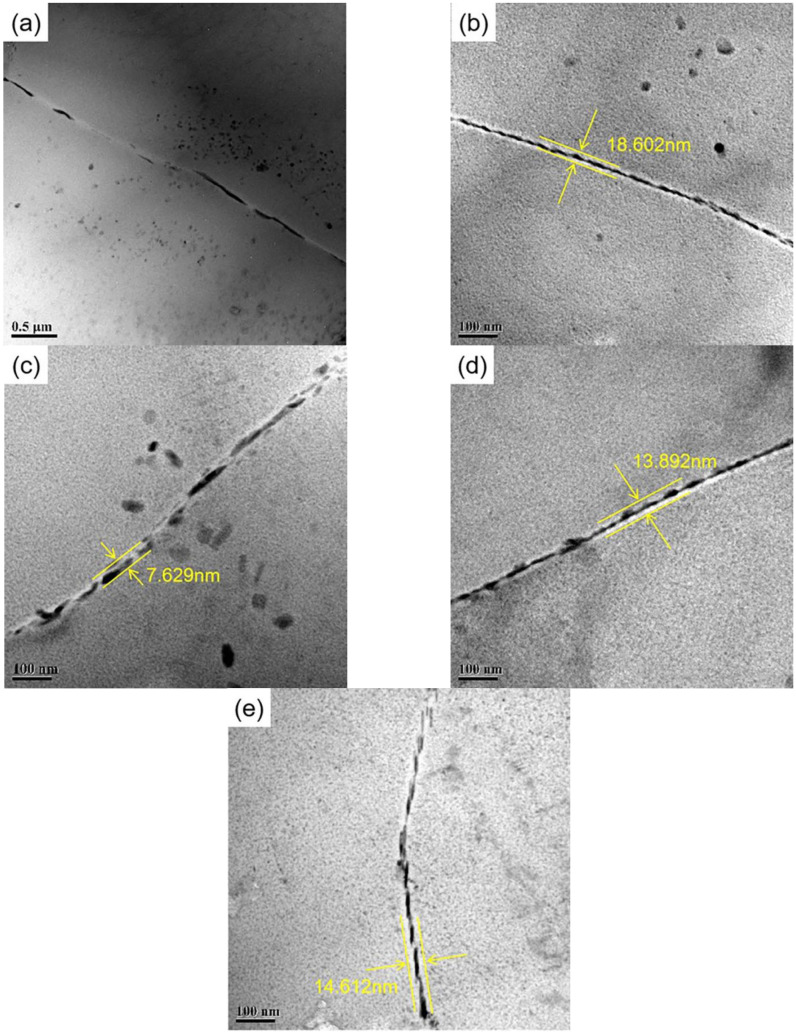
Bright field images at GB of alloy under different states: (**a**) S0; (**b**) SF; (**c**) S135; (**d**) S270; (**e**) S450.

At the same time, the width of the precipitation-free zone (PFZ) observed here also changes greatly. After peak treatment, the precipitated phase is distributed continuously along the grain boundary. The width of the PFZ is approximately 18.6 nm, as shown in Figure 10b. After the applied stress, it can be observed that the grain boundary precipitates also grow and distribute discontinuously along the grain boundary. At the same time, it can be found that the coarsened GBP also grew and distributed discontinuously along the grain boundaries. Furthermore, the PFZ widths in alloys subjected to stress aging with S135 (Figure 10c), S270 (Figure 10d), and S450 (Figure 10e) were about 7.6 nm, 13.9 nm and 14.6 nm, respectively. Applying stress below a specific threshold can enhance GBP growth and widen the PFZ. In a certain range, the width of the PFZ is negatively correlated with corrosion resistance.

After the same stress-aging treatment, some scholars have made the optimal balance of mechanical properties and corrosion resistance of the alloy through 160 °C-150 MPa-2 h. The yield strength and ultimate tensile strength reached 667MPa and 710 MPa, respectively, and the intergranular corrosion depth was 324 μm [35].

**Figure 11:**
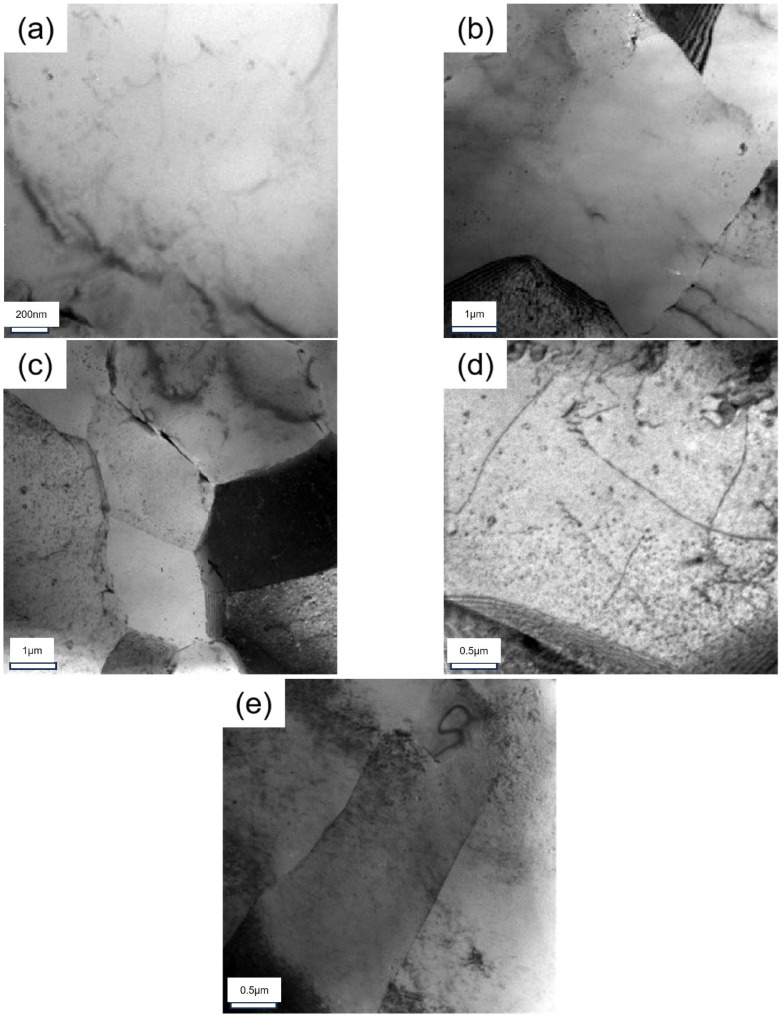
TEM images of alloys in different states: (**a**) S0; (**b**) SF; (**c**) S135; (**d**) S270; (**e**) S450.

The interaction between the precipitation phase and the deformation dislocation is observed to occur under conditions of applied stress and elevated temperature. In this effect, nailing can limit deformation and promote nucleation growth. Figure 11 shows the presence of some dislocations that become entangled as the stress increases. The density of dislocations exhibits an upward trend in response to an increase in aging stress.

## 4. Discussion

### 4.1. Aging Stress Field Affects Sample Strengthening Mechanism

After aging treatment, the mechanical properties will change. This phenomenon is primarily associated with the size, type, and morphology of the precipitated phases. Therefore, the changes in precipitates after different aging treatments lead to noticeable differences in the macroscopic properties of the materials. It is widely accepted that 7xxx aluminum alloys exhibit a significant presence of solute atoms and regions of vacancy segregation, known as Guinier–Preston (GP) zones, within the initial matrix during the early phases of the aging process. As the aging time increases, the precipitates coarsen. With the aging process, different growth states will occur in the GP region—some will be further coarsened, while others will be transformed into metastable η’ phase. With further aging, these metastable phases η’ grow and transform into stable η phases—the main strengthening-phase GP region in the material—and metastable η’ phases. Therefore, as the number of η’ phases decreases, the mechanical properties of the specimen decrease.

With the increase in applied stress in the aging process, the number of MPs increases significantly and their average size decreases overall. As shown in Figure 7, Figure 8 and Figure 9. The enhancement of material strength attributed to S0 is constrained by the limited quantity of precipitated phases, which leads to suboptimal mechanical properties. However, following the SF aging treatment, a significant number of strengthening η phases emerged, and the increase in the quantity of these precipitated phases was instrumental in enhancing the YS and UTS of the samples. Compared with SF, after the aging of S135, S270, and S450, the number of these fine needle-like and equiaxed precipitates gradually increases, resulting in a steady improvement in the performance of the sample. Based on the known classical nucleation theory, the expressions of critical nuclear energy ($W_{R}\left(R^{*}\right)$) and nucleation rate ($\dot{N}$) are shown in Equations (1) and (2) [36,37]

(1) $$W_{R}\left(R^{*}\right)=\frac{16\pi }{3}\frac{\gamma^{3}}{{\left(\Delta F_{c}+\Delta E_{e}\right)}^{2}}$$

(2) $$\dot{N}=Nv\exp\left(-\frac{W_{R}\left(R^{*}\right)}{kT}\right)\left(-\frac{Q}{kT}\right)$$

In Equation (1), *γ*, Δ*F_c_* and Δ*E_e_* are interfacial energy, volumetric free energy difference and elastic strain energy, respectively. In Equation (2), *N*, *v* and *Q,* respectively, represent the number of atoms per unit volume [38], atomic vibration frequency and diffusion activation energy in the phase. Precipitation nucleation is mainly achieved by lowering the energy barrier required for nucleation, which is achieved by introducing defects. Therefore, nucleation requires a certain energy fluctuation, and the existence of defects such as dislocation will change the free energy difference due to its high distortion energy, so the dislocation introduced by stress aging is relatively easy to nucleate. In the non-stress state, the nucleation sites inside the alloy, such as dislocation defects, are relatively limited. With the increase in applied stress, the number of introduced dislocations increases, and the energy required for nucleation decreases. Meanwhile, the increase in nucleation sites is conducive to the nucleation of MPs [39]. In addition, the solute atoms and vacancy in the sample are saturated by the solution treatment, and the interaction of the elastic stress/strain field in the dislocation makes the migration of solute atoms and vacancy easier, so the solute atoms and vacancy are more inclined to migrate to the dislocation [40]. The results show that the precipitated phase is more easily transformed into a small η’ phase under external stress, and the volume of the precipitated phase is smaller and greater than that under a non-stress state.

After stress-aging treatment, the YS, UTS, and EI of the sample are the highest when an external stress of 450 MPa is applied, and the MPs are obviously coarse at this point (Figure 7 and Figure 8). In addition, dislocation diffusion caused by the introduction of external stress early in aging may lead to intense competition among high-density MPs, which will accelerate the consumption of solute atoms. However, under the fixed alloy composition, the total number of solute atoms around MPs is fixed, and the lack of solute atoms inhibits the transition from the coarsening η’ phase to the η phase. Therefore, the average size and number of MPs of the sample under stress-aging conditions increase compared with that under non-stress conditions, which is beneficial to the improvement of the mechanical properties of the sample. Furthermore, the increased density of precipitates is due to the changes in precipitation thermodynamics and kinetics during the application of stress [41]. On the one hand, since the atomic size of Al atoms is smaller than that of Zn atoms and Mg atoms, the lattice distortion of Zn atoms and Mg atoms may occur under tensile stress, leading to a decrease in solid solubility; thus, clusters/GP zones are further inhibited from dissolving in the sample, and precipitates are more likely to form. On the other hand, the density of precipitates is also related to the coarsening kinetics of precipitates. When uniaxial stress is applied, the applied stress may cause the crystal to deform, which changes the activation energy (Q) of vacancy migration. The diffusion coefficient (*D*) of vacancy is related to the activation energy and can be expressed as [41]

(3) $$D=D_{0}\exp(-\frac{Q}{RT})$$

In Equation (3), *R* is the molar gas constant, *T* is the temperature and $D_{0}$ is the diffusion constant. *D* decreases as *Q* increases, because vacancy diffusion requires more energy to overcome. *Q* can be written as [41]:

(4) $$Q=H_{v}^{f}+H_{v}^{m}$$

where vacancy formation energy ($H_{V}^{f}$) and migration energy ($H_{v}^{m}$) are shown in Equation (4). Feng et al. [42] proposed a high-flux formula to calculate the vacancy formation energy and migration energy of uniaxial strain lattices in face-centered cubic metals. The findings indicate that the diffusion coefficient diminishes in response to applied stress. Consequently, the imposition of external stress serves to limit vacancy diffusion, impede the growth of MPt, and elevate the density of MP. In conclusion, the existence of a substantial number of fine precipitates within the crystal structure is highly advantageous for enhancing the mechanical properties of the sample, and stress aging is identified as an effective method for improving the properties of samples.

### 4.2. Aging Stress Field Affects the Corrosion Resistance Mechanism of the Sample

In this study, intergranular corrosion testing and potentiodynamic polarization were used to systematically investigate the effect of stress aging on the corrosion resistance of the sample. The corrosion sensitivity of the 7xxx aluminum alloy is primarily attributed to the microstructure at the grain boundary. It is widely recognized that the size, morphology and distribution of GBPs and PFZs significantly influence the corrosion properties of materials [40] Researchers have identified two primary corrosion mechanisms in Al-Zn-Mg-Cu alloys, namely the anodic dissolution of active grain boundary precipitates (GBPs) [43,44] and hydrogen cracking [11,44]. The presence of a localized electrochemical reaction, along with the strong electrochemical activity and high density of GBPs, accelerates the reaction, leading to corrosion [45,46,47]. However, numerous studies have also shown that a larger GBP size and varying spacing can improve the corrosion resistance of materials [48,49,50].

As aging progresses, some Zn atoms in the stable η phase at the grain boundary are replaced by Al and Cu atoms [35]. This process enriches Mg and Zn atoms, making these stable phases more corrosive because they are distributed near the grain boundary. Once the corrosion initiates, it tends to propagate from the grain boundary. In order to prevent such corrosion, a common method is to make the potential difference between GBPs and Al matrix smaller, thereby reducing the electrochemical activity of GBPs and inhibiting corrosion. As mentioned previously, increasing the content of Cu atoms and decreasing the content of Zn atoms can enhance the corrosion resistance. Furthermore, the diffusion after corrosion is also affected by the continuity of GBPs, and along with the coarsening and discontinuous growth of GBPs, the solute atoms near the grain boundary are continuously depleted, resulting in the formation of a PFZ—a region of solute depletion that inhibits the dissolution of the anode. Therefore, with the further increase in PFZ width, the corrosion situation will also be improved. Consequently, the presence of discontinuous coarse precipitates at the grain boundary, along with an appropriately sized precipitation-free zone, can effectively improve the corrosion performance of the sample.

In the polarization curve test, Icorr first increases with the aging process and then gradually decreases after the application of external stress. Compared with the SF aging state, Ecorr will increase after a decreasing process. The application of external stress leads to an increase in dislocations within the alloy, so the vacancy formed after quenching of the material and the solute atom cluster will be polymerized at the site or at the grain boundary. This alters the diffusion rate of solute atoms at the grain boundary. Thus, the growth and coarsening of grain boundary precipitates are promoted, and the GBP becomes dispersed and discontinuous. This discontinuous distribution reduces the electrochemical activity of GBPs, so the discontinuous distribution of GBPs will reduce the Icorr and increase the Ecorr.

As shown in Figure 10, in the S0, there is little grain boundary precipitation, there is a large distance between precipitates, and the PFZ width is extremely narrow. After peak-aging treatment, a large number of GBPs appear and are continuously distributed. However, during the aging process of S135, S270 and S450, the precipitates appear to have a discontinuous distribution. According to the depletion mechanism of vacancy and solute atoms [51,52], vacancy and solute atoms will migrate to the grain boundary and be consumed under the action of applied stress. When the applied stress is 135 MPa, lattice defects will appear under low external stress, resulting in a decline in solid solubility, which further inhibits the dissolution of clusters/GP zones in the sample and promotes the nucleation of new precipitates. Concurrently, the applied stress will impede the diffusion of vacancies, thus reducing the diffusion of solute atoms to the grain boundary. The grain boundary precipitation exhibits an intermittent distribution. As the applied stress increases to 270 MPa, a significant number of dislocations are introduced into the matrix. The presence of dislocations will dissolve the vacancy, thus reducing the precipitation site in the crystal. Meanwhile, the increase in applied stress also reduces the diffusion inhibition of the vacancy, so enough solute atoms migrate to the grain boundary, resulting in a relatively continuous precipitates at the grain boundary. When the applied stress increases to 450 MPa, more dislocation entangling is introduced, which reduces the degree of inhibition exerted by stress on vacancy diffusion. This results in a greater migration of vacancy and solute atoms to the grain boundary. However, the total amount of solute atoms is fixed, and more nucleation sites but not enough solute atoms lead to the reappearance of an intermittent distribution of precipitated phase at the grain boundary. The considerable impact of stress aging and peak aging on the grain boundary characteristics of the sample is shown in Figure 11.

In this study, it was found that the corrosion resistance of the alloy exhibits a strengthening trend with the application of external stress. The alloy demonstrated optimal corrosion resistance when subjected to a stress level of 135 MPa. This improved corrosion resistance can be attributed to the fact that crystal lattice defects will appear in the stress-aging state, resulting in the decline of solid solubility. At the same time, the external stress also restricts the movement of vacancy–atomic clusters. At low stress levels, this effect is more significant, combined with the competitive relationship between precipitated phases. The corrosion resistance of the Al-Zn-Mg-Cu alloy is improved by decreasing the vacancy migration to the grain boundary and the number of solute atoms.

**Figure 12:**
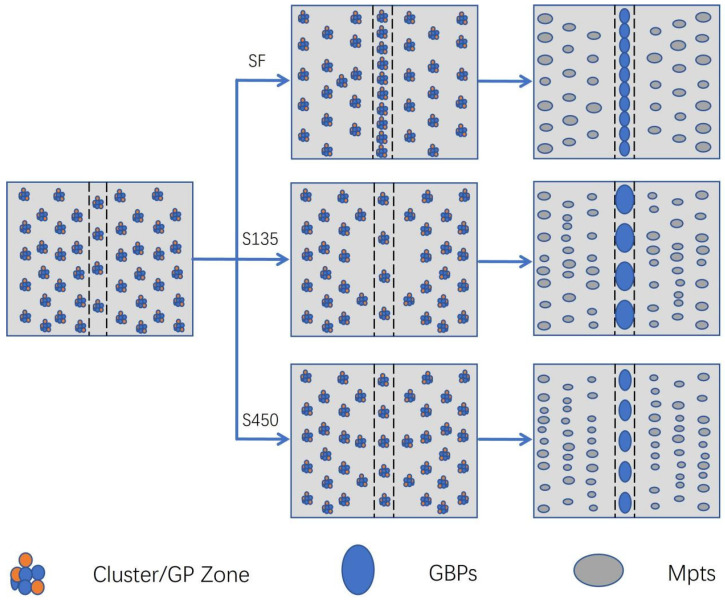
Schematic diagram of significant effects of stress aging and peak aging on grain boundary characteristics of Al-Zn-Mg-Cu alloys.

As the applied stress increases, the dislocation density in the matrix significantly rises, and even dislocation entanglement appeared. This is shown in Figure 12. A large amount of dislocation accumulation reduces the activation energy of aging precipitates in Al-Zn-Mg-Cu alloys, making the nucleation of precipitates easier. Moreover, dislocation also provides a channel for the migration of solute atoms, thus facilitating the nucleation and growth of GBP. At a stress level of 270 MPa, the increased dislocation density reduces the ability of external stress to restrict the formation of vacancy–atom clusters, allowing more solute atoms to migrate to the grain boundaries. This results in an increase in nucleation sites at the grain boundaries, causing the precipitation phase to be more continuously distributed along the grain boundaries, which in turn decreases the material’s corrosion resistance compared to that at 135 MPa. When the stress increases further to 450 MPa, the restriction ability of the applied stress on the vacancy–atomic cluster is further reduced, but the solid solubility of the solute atoms decreases. This competitive effect leads to a discontinuous distribution of precipitates at the grain boundary once again. A large number of nucleation growth near the grain boundary also consumes many solute atoms, leading to the formation and growth of PFZ. Therefore, the application of external stress can result in a large, discontinuous distribution of grain boundary precipitates and promote the formation of PFZs, which can improve the corrosion resistance of the material to a certain extent.

## 5. Conclusions

This research primarily investigates the changes in mechanical properties, corrosion resistance, and microstructure of the Al-Zn-Mg-Cu alloy following aging under applied external stress. Additionally, it examines the mechanisms through which aging stress influences the properties of the Al-Zn-Mg-Cu alloy. The principal findings are summarized as follows:

(1) The mechanical properties of the alloy improve progressively with the increase in the applied stress, though its ductility decreases. Both the yield strength (YS) and ultimate tensile strength (UTS) reach the peak values (YS: 713 MPa, UTS: 740 MPa) when the applied stress is 450 MPa. In the absence of stress, the YS and UTS are 640 MPa and 691 MPa, respectively. This is due to the external stress inducing alloy lattice defects, reducing the solubility of solute atoms, which combine with matrix precipitates (MPts) to produce a strong competitive relationship, and inhibiting the coarsening of MPts and the η’ → η phase transition, thereby improving the mechanical properties of the alloy.
(2) With the application and gradual increase in external stress, the overall Icorr of the alloy shows a downward trend. The Icorr values for stress conditions of SF, S135, S270 and S450 are 1.508 × 10^−4^, 2.962 × 10^−6^, 1.999 × 10^−5^ and 8.892 × 10^−6^, respectively. The alloy achieves the balance of mechanical properties and corrosion resistance when the applied stress is 270 MPa. This is because the applied stress limits the motion of the vacancy and solute atoms. This results in the rapid growth of a grain boundary precipitated phase (GBP) and the formation of a precipitation-free zone (PFZ). When a discontinuous GBP is formed, corrosion can be prevented to a certain extent, while a narrower PFZ within a certain range will also improve the corrosion resistance of the alloy. This phenomenon is more obvious at lower stresses.
(3) The improvement of mechanical properties is inevitably accompanied by the decrease in elongation. When the applied stress is 270 MPa, the strength, toughness and corrosion resistance of the alloy reach a relative balance in which the mechanism study of stress on mechanical properties and corrosion resistance can guide the engineering application of the material, making its application field more extensive.

## Figures and Tables

**Table 1:** Chemical composition of 7xxx aluminum alloy (wt.%).

Mg	Si	Cu	Zn	Zr	Fe	Al
3.0	0.03	3.0	10.0	0.15	0.06	Bal.

**Table 2:** Experimental protocols determined in this study.

Sample	Solution Treatment	Aging Parameter		
		Aging Time/h	Aging Temperature/°C	Aging Stress/MPa
S0	475 °C × 3 h	0	0	0
SF		24	120	0
S135		24	120	135
S270		24	120	270
S450		24	120	450

**Table 3:** Tensile properties of Al-Zn-Mg-Cu alloys under different aging conditions.

Sample	Yield Strength/MPa	Ultimate Tensile Strength/MPa	Elongation/%
SF	640	691	6.1%
S135	637	710	5.2%
S270	695	733	4.52%
S450	713	740	2.56%

**Table 4:** Electrochemical corrosion parameters obtained from the polarization curve in Figure 6.

Sample	Ecorr/V	Icorr/(A∙cm^−2^)
S0	−0.762	2.583 × 10^−5^
SF	−0.737	1.508 × 10^−4^
S135	−0.894	2.962 × 10^−6^
S270	−1.178	1.999 × 10^−5^
S450	−0.781	8.892 × 10^−6^

## Data Availability

Data will be made available on request. Contact the author if necessary.

## References

[1] Li B., Wang X., Chen H., Hu J., Huang C., Gou G.Q. (2016). Influence of heat treatment on the strength and fracture toughness of 7N01 aluminum alloy. J. Alloys Compd..

[2] Cao F.H., Zheng J.X., Jiang Y., Cheng B., Wang Y.R., Hu T. (2019). Experimental and DFT characterization of η′ nano-phase and its interfaces in Al-Zn-Mg-Cu alloys. Acta Mater..

[3] Yang X.B., Chen J.H., Liu J.Z., Qin F., Xie J., Wu C.L. (2014). A high-strength Al-Zn-Mg alloy hardened by the T-phase precipitates. J. Alloys Compd..

[4] Li Z.M., Jiang H.C., Wang Y.L., Zhang D., Yan D.S., Rong L.J. (2018). Effect of minor Sc addition on microstructure and stress corrosion cracking behavior of medium strength Al-Zn-Mg alloy. J. Mater. Sci. Technol..

[5] Wang Y.L., Jiang H.C., Li Z.M., Yan D.S., Zhang D., Rong L.J. (2018). Two-stage double peaks ageing and its effect on stress corrosion cracking susceptibility of Al-Zn-Mg alloy. J. Mater. Sci. Technol..

[6] Ren X., Zhang J., Zhang Z., Wang Q., Xue Y., Liu H., Meng M., Zhao X., Liu H. (2024). Influence of the solid solution duration on the microstructure and mechanical properties of the Al-10.0Zn-3.0Mg-2.5Cu alloy. Mater.

[7] Fu Z.L., Zhao X., Jiao M.H., Ren X.W., Zhao H.B., Liu H.L. (2024). Study on the Aging Precipitation Behavior and Kinetics of Al-10.0Zn-3.0Mg-2.8Cu Alloy by Pre-Deformation Treatment. Mater.

[8] Xiao F., Shu D., Wang D.H., Zhu G.L., Wang S.B., Sun B.D. (2023). Effect of Zn content on the formability and aging precipitation of Al-Zn-Mg-Cu-Nb alloys prepared by LPBF. J. Mater. Sci. Technol..

[9] Liu X.Y., Pan Q.L., Zhang X.L., Liang S.X., Zheng L.Y., Gao F., Xie H.L. (2014). Effects of stress-aging on the microstructure and properties of an aging forming Al-Cu-Mg-Ag alloy. Mater. Des..

[10] Guo W., Guo J., Wang J., Yang M., Li H., Wen X., Zhang J. (2015). Evolution of precipitate microstructure during stress aging of an Al–Zn–Mg–Cu alloy. Mater. Sci. Eng. A.

[11] Wang R., Wang D.T., Nagaumi H., Wu Z.B., Zhang X.Z., Li X.Z. (2022). Effect of Zn content on corrosion resistance of as-cast Al-6Si-3Cu alloy. Mater. Lett..

[12] Wang Y.Z., Shi L., Su R.M., Liu T.Y., Li G.L. (2024). Effect of Variable Rate Non-isothermal Aging on the Microstructure and Properties of Al-Zn-Mg-Cu Alloy. JOM.

[13] Zhu Z., Jiang X., Wei G., Fang X., Zhong Z., Song K., Han J., Jiang Z. (2020). Influence of Zn Content on Microstructures, Mechanical Properties and Stress Corrosion Behavior of AA5083 Aluminum Alloy. Acta Metall. Sin..

[14] Wei Q.Y., Zhao Y.L., Liu H., He W.X., Wang M.M., Sun Z.Z. (2024). Effect of heat treatment on the deformation behavior of an Al-Cu alloy studied by in-situ synchrotron X-ray diffraction. Mater.

[15] Li Y., Xu G.F., Peng X.Y., Wang F.X., Deng Y., Liang X.P. (2020). Effect of different aging treatment on high temperature properties of die-forged Al-5.87Zn-2.07Mg-2.42Cu alloy. Mater. Charact..

[16] Yu Z., Li H., Cai P., Fu X., Feng Z., Zhang L., Wang J., Xiao N. (2023). Effect of aging route on the precipitation behavior and thermal stability of Al-Cu-Mg-Ag alloy. J. Mater. Sci. Technol..

[17] Xiong Y., Robson J.D., Cao Z., Deng Y., Yao Y., Zhong X., Bendo A., Jinlong L., Guarracino F., Donoghue J. (2023). Mitigation effects of over-aging (T73) induced intergranular corrosion on stress corrosion cracking of AA7075 aluminum alloy and behaviors of η phase grain boundary precipitates during the intergranular corrosion formation. Corros. Sci..

[18] Liu L., Hou Y., Ye T., Zhang L., Huang X., Gong Y., Liu C., Wu Y., Duan S. (2024). Effects of Aging Treatments on the Age Hardening Behavior and Microstructures in an Al-Mg-Si-Cu Alloy. Metals.

[19] Sanyal S., Chatterjee S., Chabri S., Bhowmik N., Sinha A. (2019). Influence of over-aging and sub-zero temperature rolling on strength-ductility balance in AA6061 alloy. Eng. Sci. Technol. Int. J..

[20] Ren J., Wang R., Peng C., Zhang H., Xu C., Wu Y., Feng Y. (2020). Effect of repetitious retrogression and re-aging treatment on the microstructure, strength and corrosion behavior of powder hot-extruded 7055 Al alloy. Mater. Charact.

[21] Xie P., Chen S., Chen K., Jiao H., Huang L., Zhang Z., Yang Z. (2019). Enhancing the stress corrosion cracking resistance of a low-Cu containing Al-Zn-Mg-Cu aluminum alloy by step-quench and aging heat treatment. Corros. Sci..

[22] Su H., Toda H., Shimizu K., Uesugi K., Takeuchi A., Watanabe Y. (2019). Assessment of hydrogen embrittlement via image-based techniques in Al-Zn-Mg-Cu aluminum alloy. Acta Mater..

[23] Guo X.B., Zhang J., Deng Y.L., Zhang X.M., Zhang Y. (2019). Effect of grain boundaries on the preferential orientation distribution of θ′ precipitates in stress-aged Al-2Cu alloy bicrystals. J. Alloys Compd..

[24] Wu X.T., Zhan L.H., Guan C.L., Yang X.B., He J.Y. (2019). Effect of creep aging parameters on creep resistance behavior of Al-Cu-Mg alloy. Mater. Res. Express.

[25] Chen F., Zhan L.-H., Xu Y.-Q., Liu C.-H., Ma B.-L., Zeng Q.-Q., Hu Z.-G., Zhu W.-L., Yan D.-Y. (2024). Regulation mechanism of aging behavior and mechanical properties of 2195-T34 Al-Li alloy at different stress levels. J. Cent. S. Univ..

[26] Zhu A.W., Starke E.A. (2001). Stress aging of Al-xCu alloys: Experiments. Acta Mater..

[27] Guo W., Yang M., Zheng Y., Zhang X., Li H., Wen X., Zhang J. (2013). Influence of elastic tensile stress on aging process in an Al-Zn-Mg-Cu alloy. Mater. Lett..

[28] Guo X.B., Zhang J., Chen J.Q., Deng Y.L. (2020). Effect of edge dislocations on the distribution of θ′ precipitates in stress-aged Al-Cu single crystal. J. Alloys Compd..

[29] Quan L.-W., Zhao G., Tian N., Huang M.-L. (2013). Effect of stress on microstructures of creep-aged 2524 alloy. Trans. Nonferrous Met. Soc. China.

[30] Lin Y.C., Zhang J.L., Chen M.S. (2016). Evolution of precipitates during two-stage stress-aging of an Al-Zn-Mg-Cu alloy. J. Alloys Compd..

[31] Lin Y.C., Peng X.B., Jiang Y.Q., Shuai C.J. (2018). Effects of creep-aging parameters on aging precipitates of a two-stage creep-aged Al-Zn-Mg-Cu alloy under the extra compressive stress. J. Alloys Compd..

[32] Zhang D., Jiang H.C., Cui Z.J., Yan D.S., Song Y.Y., Rong L.J. (2021). Precipitation kinetics in an Al-Zn-Mg alloy during stress-aging. J. Alloys Compd..

[33] Liu Y.X., Li J.M., Gao F., Liang S.X., Zhang X.L., Cui H.X. (2015). Effects of aging treatment on the intergranular corrosion behavior of Al-Cu-Mg-Ag alloy. J. Alloys Compd..

[34] Li S., Dong H.G., Li P., Chen S. (2018). Effect of repetitious non-isothermal heat treatment on corrosion behavior of Al-Zn-Mg alloy. Corros. Sci..

[35] Zhang Z., Ma X., Zhang C., Chu G., Meng Z., Zhao G., Chen L. (2022). Effect of stress-aging treatment on the mechanical and corrosion properties of Al-Zn-Mg-Cu alloy. Mater. Sci. Eng. A.

[36] Clouet E., Nastar M., Sigli C. (2004). Nucleation of Al3Zr and Al3Sc in aluminum alloys: From kinetic Monte Carlo simulations to classical theory. Phys. Rev. B.

[37] Guo F., Duan S., Pan Y., Wu D., Matsuda K., Wang T., Zou Y. (2023). Stress corrosion behavior and microstructure analysis of Al-Zn-Mg-Cu alloys friction stir welded joints under different aging conditions. Corros. Sci..

[38] Ren Y., Wan T., Xu Y., Zhang K., Zhang M., Li J. (2024). Effects of stress aging treatment on the microstructure, mechanical properties and electrochemical corrosion behavior of Al-Zn-Mg-Cu alloy. J. Alloys Compd..

[39] Shang F.U., Yi D.Q., Liu H.Q., Jiang Y., Wang B., Hu Z. (2014). Effects of external stress aging on morphology and precipitation behavior of θ″ phase in Al-Cu alloy. Trans. Nonferrous Met. Soc. China.

[40] Zhang D., Jiang H.C., Cui Z.J., Yan D.S., Song Y.Y., Rong L.J. (2022). Synchronous improvement of mechanical properties and stress corrosion resistance by stress-aging coupled with natural aging pre-treatment in an Al-Zn-Mg alloy with high recrystallization fraction. J. Mater. Sci. Technol..

[41] Feng Y., Liu M., Shi Y., Ma H., Li D., Li Y., Lu L., Chen X. (2019). High-throughput modeling of atomic diffusion migration energy barrier of fcc metals. Prog. Nat. Sci. Mater. Int..

[42] Tan P., Liu Z.Q., Qin J., Wei Q.R., Wang B., Yi D.Q. (2024). Enhanced corrosion performance by controlling grain boundary precipitates in a novel crossover Al-Cu-Zn-Mg alloy by optimizing Zn content. Mater. Charact..

[43] Zhang Y., Yang H., Huang R., Sun P., Zheng S., Li M., Wang X., Du Q. (2024). Investigation of microstructure and corrosion resistance of an Al-Zn-Mg-Cu alloy under various ageing conditions. Corros. Sci..

[44] Tang J., Wang Y., Fujihara H., Shimizu K., Hirayama K., Ebihara K., Takeuchi A., Uesugi M., Toda H. (2024). Stress corrosion cracking induced by the combination of external and internal hydrogen in Al-Zn-Mg-Cu alloy. Scr. Mater..

[45] Song F.X., Zhang X.M., Liu S.D., Tan Q., Li D.F. (2014). The effect of quench rate and overageing temper on the corrosion behaviour of AA7050. Corros. Sci..

[46] Sun X., Zhang B., Lin H., Zhou Y., Sun L., Wang J., Han E.-H., Ke W. (2013). Correlations between stress corrosion cracking susceptibility and grain boundary microstructures for an Al-Zn-Mg alloy. Corros. Sci..

[47] Knight S.P., Birbilis N., Muddle B.C., Trueman A.R., Lynch S.P. (2010). Correlations between intergranular stress corrosion cracking, grain-boundary microchemistry, and grain-boundary electrochemistry for Al-Zn-Mg-Cu alloys. Corros. Sci..

[48] Xiao Q., Xu Y., Huang J., Li B., Wang B., Liu S., Fu L. (2020). Effects of quenching agents, two-step aging and microalloying on tensile properties and stress corrosion cracking of Al-Zn-Mg-Cu alloys. J. Mater. Sci. Technol..

[49] Qiu Y., Liu R., Zou L., Chi H., Wang C., Wang B., Chen J. (2022). Influence of Grain Boundary Precipitates on Intergranular Corrosion Behavior of 7050 Al Alloys. Coatings.

[50] Metalnikov P., Ben-Hamu G., Shin S.K. (2019). Relation Between Zn Additions, Microstructure and Corrosion Behavior of New Wrought Mg-5Al Alloys. Met. Mater. Int..

[51] Li Z.G., Chen L., Tang J.W., Zhao G.Q., Zhang C.S. (2020). Response of mechanical properties and corrosion behavior of Al-Zn-Mg alloy treated by aging and annealing: A comparative study. J. Alloys Compd..

[52] Zhang Y., Jin S., Trimby P.W., Liao X., Murashkin M.Y., Valiev R.Z., Liu J., Cairney J.M., Ringer S.P., Sha G. (2019). Dynamic precipitation, segregation and strengthening of an Al-Zn-Mg-Cu alloy (AA7075) processed by high-pressure torsion. Acta Mater..

